# Supplementary material for: Education for Sustainable Development (ESD) in Romanian Higher Education Institutions (HEIs) within the SDGs Framework
Source: Int J Environ Res Public Health. 2022 Feb 11;19(4):1998. doi: 10.3390/ijerph19041998 (PMC8872118; doi:10.3390/ijerph19041998)
Supplement: Supplementary file 1 [file ijerph-19-01998-s001.zip › ijerph-1561246-supplementary.pdf]

International Journal of
Environmental Research and Public Health

MDPI

Supplementary

Interview Script

Date____________

Taking into consideration the definition of UNESCO on ESD, please answer the questions below as accurate as possible. Thank you kindly.

According to UNESCO, "Education for Sustainable Development develops and strengthens the capacity of individuals, groups, communities, organizations and countries to make judgements and choices in favour of sustainable development. It can promote a shift in people's mind-sets and in so doing enable them to make our world safer, healthier and more prosperous, thereby improving the quality of life. Education for sustainable development can provide critical reflection and greater awareness and empowerment so that new visions and concepts can be explored, and new methods and tools developed" (Unesco, 2005, p. 1).

1. Please specify your position within the HEI ____________________________________

General ESD aspects

2. Does your HEI ever signed any Declaration, Charter or Initiative connected to ES or ESD?
   - Yes
   - No
   - I don't know/ I don't want to answer

If yes, which Declaration, Charter or Initiative?

____

3. Did your HEI adopt ESD in their policies?
   - Yes
   - No
   - I don't know/ I don't want to answer

If yes, please offer some details.

____

____

4. Did your HEI adopt ESD in their mission/ vision/ goals/objectives?
   - Yes
   - No
   - I don't know/ I don't want to answer

If yes, please offer some details.

5. Did your HEI adopt ESD in their organization (departments/offices)?
    - Yes
    - No
    - I don't know/ I don't want to answer

If yes, please offer some details.

6. Did/does your HEI have a Strategic Plan for ESD?
    - Yes
    - No
    - I don't know/ I don't want to answer

If yes, please offer some details.

7. Are there staff members dedicated for ESD?
    - Yes
    - No
    - I don't know/ I don't want to answer

If yes, please offer some details.

8. Does your HEI have a dedicated ESD department?
    - Yes
    - No
    - I don't know/ I don't want to answer

If yes, please offer some details.

9. Do your HEI leaders support ESD by providing funds or other type of support?
    - Yes
    - No
    - I don't know/ I don't want to answer

If yes, please offer some details.

Campus operations

10. Digitalisation

    Yes

    No

    I don't know/ I don't want to answer

If yes, please offer some details.

11. Waste reduction

    Yes

    No

    I don't know/ I don't want to answer

If yes, please offer some details.

12. Access and facilities for disabled people

    Yes

    No

    I don't know/ I don't want to answer

If yes, please offer some details.

13. Waste bins for separation

    Yes

    No

    I don't know/ I don't want to answer

If yes, please offer some details.

14. Alternative energy

    Yes

    No

    I don't know/ I don't want to answer

If yes, please offer some details.

15. Energy reduction

    Yes

    No

    I don't know/ I don't want to answer

If yes, please offer some details.

16. Building operations

Yes

No

I don't know/ I don't want to answer

If yes, please offer some details.

17. Water management

Yes

No

I don't know/ I don't want to answer

If yes, please offer some details.

Please feel free to add any other Campus operations promoted by your HEI as far as SD is concerned.

Education (sustainability incorporated curriculum/ pedagogy)

18. Sustainability incorporated curriculum for all students

Yes

No

I don't know/ I don't want to answer

If yes, please offer some details.

19. Sustainability (optional) incorporated curriculum for all students

Yes

No

I don't know/ I don't want to answer

If yes, please offer some details.

20. SD major for Bachelor's level

Yes

No

I don’t know/ I don’t want to answer

If yes, please offer some details.

21. SD major for Master's level

Yes

No

I don’t know/ I don’t want to answer

If yes, please offer some details.

22. Role plays, simulation, discussions and debates on SD

Yes

No

I don’t know/ I don’t want to answer

If yes, please offer some details.

23. SD major for PhD's level

Yes

No

I don’t know/ I don’t want to answer

If yes, please offer some details.

24. SD training for lectures/professors on SD

Yes

No

I don’t know/ I don’t want to answer

If yes, please offer some details.

25. Multiple teaching-learning methodologies for teaching sustainability

Yes

No

I don’t know/ I don’t want to answer

If yes, please offer some details.

26. Possibility for students to take classes in other faculty (by modules)

    Yes

    No

    I don't know/ I don't want to answer

If yes, please offer some details.

27. Invited lectures/professors on SD

    Yes

    No

    I don't know/ I don't want to answer

If yes, please offer some details.

28. SD internships

    Yes

    No

    I don't know/ I don't want to answer

If yes, please offer some details.

Please feel free to add any other measures concerning Education (sustainability incorporated curriculum/ pedagogy) promoted by your HEI as far as SD is concerned.

Research

29. Researchers' Platform

    Yes

    No

    I don't know/ I don't want to answer

If yes, please offer some details.

30. Green Projects on SD

    Yes

    No

I don’t know/ I don’t want to answer

If yes, please offer some details.

31. Encourage and promote joint research

Yes

No

I don’t know/ I don’t want to answer

If yes, please offer some details.

32. Research Centre on SD

Yes

No

I don’t know/ I don’t want to answer

If yes, please offer some details.

33. Funding SD Research

Yes

No

I don’t know/ I don’t want to answer

If yes, please offer some details.

34. Use of research generated in SD teaching

Yes

No

I don’t know/ I don’t want to answer

If yes, please offer some details.

35. Publications on SD

Yes

No

I don’t know/ I don’t want to answer

If yes, please offer some details.

36. Patents in SD

Yes

No

I don't know/ I don't want to answer

If yes, please offer some details.

Please feel free to add any other measures concerning Research promoted by your HEI as far as SD is concerned.

Community outreach and collaborations

37. SD Exchange programmes

Yes

No

I don't know/ I don't want to answer

If yes, please offer some details.

38. Joint SD degrees with other HEIs

Yes

No

I don't know/ I don't want to answer

If yes, please offer some details.

39. Joint SD research with other HEIs

Yes

No

I don't know/ I don't want to answer

If yes, please offer some details.

40. Collaboration in SD research projects

Yes

No

I don't know/ I don't want to answer

If yes, please offer some details.

41. SD partnerships with other stakeholders

Yes

No

I don’t know/ I don’t want to answer

If yes, please offer some details.

42. Part of a UN Regional Centre of Expertise (RCE)

Yes

No

I don’t know/ I don’t want to answer

If yes, please offer some details.

43. Part of interdisciplinary SD expert networks

Yes

No

I don’t know/ I don’t want to answer

If yes, please offer some details.

44. Academic staff involved in voluntary advisory activities in SD

Yes

No

I don’t know/ I don’t want to answer

If yes, please offer some details.

45. SD events that are open to the public/community

Yes

No

I don’t know/ I don’t want to answer

If yes, please offer some details.

Please feel free to add any other measures concerning Community outreach and collaborations promoted by your HEI as far as SD is concerned.

On-campus experience

46. Involvement of students in SD activities
    - Yes
    - No
    - I don’t know/ I don’t want to answer

If yes, please offer some details.

---

47. Involvement of professors and researchers in SD activities
    - Yes
    - No
    - I don’t know/ I don’t want to answer

If yes, please offer some details.

Please feel free to add any other measures concerning On-campus experience promoted by your HEI as far as SD is concerned.

Barriers against ESD adoption with Romanian HEIs

48. Lack of awareness:

	Yes	No	I don’t know/ I don’t want to answer
Ignorance and misunderstandings of SD concept			
Lack of leadership support			
Lack of policies for SD support			
Lack of responsibility towards SD			
SD perceived as unimportant for the curriculum			

49. Lack of resources:

	Yes	No	I don’t know/ I don’t want to answer
Funding			
Lack of staff and more experienced officers to adopt SD			
Infrastructure			
Priorities			
Government policies			

50. Structural deficiencies:

	Yes	No	I don’t know/ I don’t want to answer
Curriculum not adapted for SD			

Lack of management support as well as infrastructure to ensure SD			
Lack of communication (leaders / staff)			
Conservative leadership			
Resistance to change			
Focus on short term goals			

Please feel free to add any other Barriers against ESD adoption with Romanian HEIs.

____

____

HEI code	Interview type	Interview date
HEI1	Zoom	3 September 2021
HEI2	Zoom	10 September 2021
HEI3	Zoom	11 September 2021 (rescheduled)
HEI4	Skype	17 September 2021
HEI5	Zoom	24 September 2021 (rescheduled)
HEI6	Skype	30 September 2021
HEI7	Zoom	11 October 2021 (rescheduled)

Interview characteristics (code, date, type)

Positions of participants within the HEI

HEI1	Senior Researcher
HEI2	Associate Professor
HEI3	Senior Professor
HEI4	Vice-Rector
HEI5	Director of the Office of Sustainability
HEI6	Technology Transfer Office, Director
HEI7	Professor
